# Effects of Dehydration on Brain Perfusion and Infarct Core After Acute Middle Cerebral Artery Occlusion in Rats: Evidence From High-Field Magnetic Resonance Imaging

**DOI:** 10.3389/fneur.2018.00786

**Published:** 2018-09-20

**Authors:** Yuan-Hsiung Tsai, Jenq-Lin Yang, I-Neng Lee, Jen-Tsung Yang, Leng-Chieh Lin, Yen-Chu Huang, Mei-Yu Yeh, Hsu-Huei Weng, Chia-Hao Su

**Affiliations:** ^1^Departments of Diagnostic Radiology, Chang Gung Memorial Hospital, Chiayi, College of Medicine, Chang Gung University, Taoyuan, Taiwan; ^2^Institute for Translational Research in Biomedicine, Kaohsiung Chang Gung Memorial Hospital, Kaohsiung, Taiwan; ^3^Department of Medical Research, Chang Gung Memorial Hospital, Chiayi, Taiwan; ^4^Department of Neurosurgery Chang Gung Memorial Hospital, Chiayi, College of Medicine, Chang Gung University, Taoyuan, Taiwan; ^5^Department of Emergency Medicine Chang Gung Memorial Hospital, Chiayi, College of Medicine, Chang Gung University, Taoyuan, Taiwan; ^6^Department of Neurology, Chang Gung Memorial Hospital, Chiayi, College of Medicine, Chang Gung University, Taoyuan, Taiwan; ^7^Department of Biomedical Engineering and Environmental Sciences, National Tsing Hua University, Hsinchu, Taiwan

**Keywords:** dehydration, cerebral blood flow, acute stroke, MRI, middle cerebral artery occlusion

## Abstract

**Background:** Dehydration is common among ischemic stroke patients and is associated with early neurological deterioration and poor outcome. This study aimed to test the hypothesis that dehydration status is associated with decreased cerebral perfusion and aggravation of ischemic brain injury.

**Methods:** Diffusion-weighted imaging and arterial spin labeling perfusion MR imaging were performed on rats with middle cerebral artery occlusion (MCAO) by using a 9.4T MR imaging scanner to measure the volume of infarction and relative cerebral blood flow (rCBF) after infarction. Twenty-five rats were assigned to either a dehydration group or normal hydration group, and dehydration status was achieved by water deprivation for 48 h prior to MCAO.

**Results:** The volume of the infarction was significantly larger for the dehydration group at the 4th h after MCAO (*p* = 0.040). The progression in the infarct volume between the 1st and 4th h was also larger in the dehydration group (*p* = 0.021). The average rCBF values of the contralateral normal hemispheres at the 1st and the 4th h were significantly lower in the dehydration group (*p* = 0.027 and 0.040, respectively).

**Conclusions:** Our findings suggested that dehydration status is associated with the progression of infarct volume and decreases in cerebral blood flow during the acute stage of ischemic stroke. This preliminary study provided an imaging clue that more intensive hydration therapies and reperfusion strategies are necessary for the management of acute ischemic stroke patients with dehydration status.

## Introduction

Clinical guidelines suggest the assessment of volume status and emphasize the importance of adequate hydration after stroke (1). Recent studies have demonstrated that dehydration is common among stroke subjects (2, 3), and hydration status is associated with stroke-in-evolution (4, 5), discharge outcome and admission costs in acute ischemic stroke (6–8). The reasons for these observations are based on the concept that dehydration status may lead to cerebral hypoperfusion and decreased collateral blood flow, which may exacerbate the ischemic brain injury (6, 9, 10). In an animal study, Paczynski et al. reported that both extremes of hydration and dehydration spectrum would lead to more severe brain edema and midline brain structure shifting (11). However, the evidence for the effect of dehydration status on cerebral perfusion and the evolution of the ischemic core after occlusion of major arteries have, until now, never been discussed in the literature.

Magnetic resonance (MRI) diffusion-weighted imaging (DWI) identifies tissue wherein diffusion is restricted as a result of cytotoxic edema due to ischemia, which leads to a reduction in the apparent diffusion coefficient (ADC). Arterial spin-labeling (ASL) is an MRI technique that can be used to identify and quantify cerebral perfusion. It provides noninvasive, quantitative measurements of cerebral blood flow (CBF) with relative insensitivity to permeability (12).

The goal of our current study was to investigate the role of hydration status in the cerebral perfusion and infarct core using an acute middle cerebral artery occlusion (MCAO) rat model. Dehydration was achieved by water deprivation for 48 h prior to MCAO. DWI and ASL imaging were used to measure the size of infarct cores and relative cerebral blood flow (rCBF), respectively. We hypothesized that dehydration status would be associated with decreased cerebral perfusion and aggravation of ischemic brain injury.

## Methods

### Stroke animal model

Adult male Sprague-Dawley rats, weighing 250–300 g, were maintained at a constant temperature (21 ± 2°C) and humidity in a facility recognized by the Association for Assessment and Accreditation of Laboratory Animal Care International (AAALAC International) under a 12/12-h light/dark cycle with free access to food and water. All experimental procedures were approved and supervised by the Animal Committee of Chang Gung Memorial Hospital and were in compliance with the guidelines for animal care and use set forth by that Committee (Approval No.20160301803). Focal cerebral ischemia was induced via extracranial intraluminal MCAO, following the previously described method of Longa et al. (13). Briefly, the left common carotid artery, the external carotid artery (ECA) and the internal carotid artery (ICA) were sequentially exposed following anesthetization of the animal by intramuscular injection of a mixture of zoletil50 25 mg/kg and xylazine 10 mg/kg. A silicon-coated 30-mm length of 4–0 polyamide monofilament non-absorbable surgical suture was inserted via the ECA into the ICA until the bifurcation of the left middle cerebral artery (MCA) and the anterior cerebral artery in order to block the circulation in the left MCA territory. After 1 h of vessel occlusion, blood flow was restored (reperfusion) by the withdrawal of the inserted suture. The rats were then sent for the 1st MRI scan and sacrificed with carbon dioxide gas after the 2nd MRI scan that was started at the 4th h after MCAO.

### Dehydration protocols

A total of 25 Sprague-Dawley rats were randomly assigned to either the euhydration/MCAO or dehydration/MCAO group. Each rat in the dehydration group was individually housed in a metabolic cage for the 48 h prior to the MCAO surgery. Water deprivation for 48 h was accomplished by removing the water bottle from the cage (14, 15). Between the 1st and the 2nd MRI scans, rats randomized into euhydration group had free access to water while those in dehydration group were restricted from drinking water.

### MRI acquisition and analysis

MRI was performed using a 9.4-T horizontal-bore animal MR scanning system (Biospec 94/20, Bruker, Ettlingen, Germany). The MRI were started at 1 and 4 h post MCAO for each rat to generate DWI ADC and ASL CBF maps with scan time around 40 min. Before the MR imaging, animals were anesthetized with 3.0% isoflurane in room air and were placed inside the magnet. During the MRI measurements, the anesthesia delivery to the rats was maintained using a gas mixture of ~1.5-2.0% isoflurane and oxygen. Animals were physiologically monitored throughout the MR imaging experiments.

**Figure 1:**
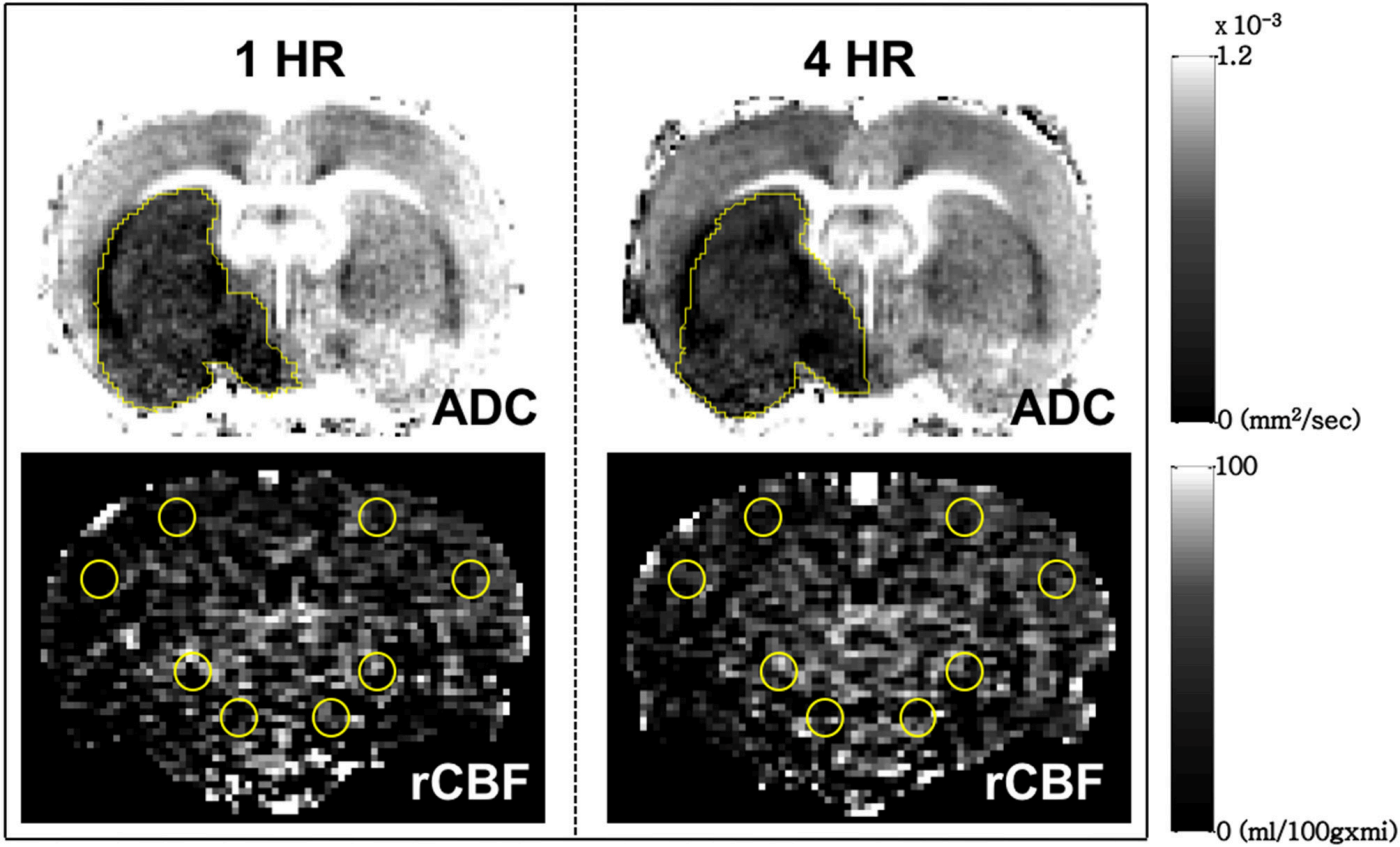
Measurement of infarct volume (**Upper row**) and relative cerebral blood flow (**Lower row**) by manual drawing regions of interest on the coronal view of the brain of a typical male Sprague Dawley rat used in this study. The relative cerebral blood flow for each hemisphere was defined as the average value measured from the 4 circular ROIs in cortical and subcortical brain regions.

DWI scans were obtained to produce ADC maps using spin-echo EPI with the following parameters: matrix = 128 × 128; field of view = 2 × 2 cm2; TR = 3000 ms; echo time (TE) = 27 ms; number of slices = 9, which were 1-mm thick with a 0.5-mm gap; three directions = x, y, z; and b values = 0, 100, 300, 500, 800, and 1,000 s/mm2. The ASL CBF images were acquired using flow alternating inversion recovery (FAIR)-echo planar imaging (EPI) in a single coronal slice (the selection of the slice was based on our experience of the slice with a maximal area of infarction) with the following parameters: matrix = 96 × 96; field of view = 2 × 2 cm^2^; spectral width = 250 kHz; repetition time (TR) = 13000 ms; echo time (TE) = 16 ms; 3-mm selected slab thickness; 1-mm slices with 4-mm labeling coverage and 25 TI values. Twenty-five pairs of images (total time 11 min and 53 s) were acquired for signal averaging. The relative CBF (rCBF) map was calculated from the ASL images using the Algebra Tool on the Bruker console. The volume of the infarction and the ADC value were manually measured on the ADC map using polygon selection on Paravision 5.1 (Bruker, Ettlingen, Germany). The rCBF was measured by manual drawing circular regions of interest (ROI) on the ASL images. Four ROIs were selected in each cerebral hemisphere, with 2 of them located in cortical regions and the others in subcortical regions. The rCBF for each hemisphere was defined as the average values measured across the 4 ROIs (Figure 1).

### Statistical analysis

All statistical analyses were performed using the Statistical Program for Social Sciences (SPSS) statistical software (version 18, Chicago, IL, USA). Variables were expressed as the mean ± standard deviation and were compared by performing Mann–Whitney U tests. The correlation between progression of infarct volume and rCBF was tested by the Person correlation. The level of statistical significance was set at *P* < 0.05.

## Results

Fifteen rats were included in dehydration group and 10 in control group. Five rats in the dehydration group died before the 1st MRI scan and other 2 died during the 1st MRI scan, thus, excluded. Three rats in control group completed the 1st MRI scan but died before the 2nd MRI scan; thus, only seven rats from the control group had data from the 2nd MRI scan. The results are shown in the Table 1. (The data of individual measurement are presented in the Supplementary Table 1 and Supplementary Figure 1). The mean ADC values of the selected areas were 456.1 and 441.6 10^−6^mm2/sec at the 1st h for euhydration and dehydration groups respectively, which confirmed the definition of tissue infarction. Although there was no significant difference between the volumes of the infarctions during the 1st h within each of the groups, the volume of the infarction was significantly larger in the dehydration group at the 4^th^ h after MCAO (*p* = 0.040) than that of the control group. The absolute volume of the infarct progression between the 1st and 4th h was also larger in the dehydration group (*p* = 0.021). There were no significant differences between the measured absolute ADC values of each group at the 2 time points. The rCBF values of the infarct side were lower in dehydration group than in the control group at the 1st and 4th h after MCAO but did not reach statistical significance. On the contrary, the rCBF values of the contralateral normal side were significantly lower in the dehydration group than in the control group at the 1st and 4th h after MCAO (*p* = 0.027 and 0.040, respectively). However, the rCBF value at the 4th h and the rCBF ratio (infarct/normal), as well as the recovery of rCBF within 4 hours, were not significantly different between the two groups. There was a negative correlation between the rCBF of normal hemisphere at the 4th h and the progression of infarct size for all rats (Person correlation, *r* = −0.628, *p* = 0.012). In subgroup analysis, there was a moderate correlation between rCBF of normal hemisphere at the 4th h and the progression of infarct size for the dehydrated rats but did not reach statistical significance (*r* = −0.628, *p* = 0.095).

**Table 1:** Comparison of infarct volume and CBF based on ADC and ASL in two groups.

	**Control (*n* = 10 for 1 h and 7 for 4 h)**	**Dehydration (*n* = 8)**	***p*-value**
Volume of infarction (mm^3^)			
1 h after MCAO	39.4 (15.3)	59.9 (46.2)	0.573
4 h after MCAO	75.9 (22.2)	105.5 (46.3)	0.040^*^
Ratio of progression	1.46	2.34	0.121
Absolute volume of progression	24.1(9.4)	45.6 (36.8)	0.021^*^
ADC value (× 10^−6^ mm^2^/sec)			
Infarct side			
1 h after MCAO	456.1 (53.6)	441.6 (54.9)	0.573
4 h after MCAO	525.5 (75.0)	449.5 (46.3)	0.867
Normal side			
1 h after MCAO	829.2 (45.6)	789.4 (40.8)	0.122
4 h after MCAO	851.5 (41.3)	816.0 (44.4)	0.694
Ratio (Infarct/Normal)			
1 h after MCAO	0.55	0.56	0.573
4 h after MCAO	0.61	0.55	0.955
Relative CBF (ml/100 g^−1^min^−1^)			
Infarct side			
1 h after MCAO	10.6 (5.2)	5.7 (2.6)	0.101
4 h after MCAO	10.9 (5.6)	7.7 (3.1)	0.397
Normal side			
1 h after MCAO	25.6 (5.5)	19.8 (4.8)	0.027^*^
4 h after MCAO	24.1 (2.8)	20.7 (2.9)	0.040^*^
Ratio (Infarct/Normal)			
1 h after MCAO	0.42	0.30	0.408
4 h after MCAO	0.44	0.37	0.613
Recovery of perfusion in 4 h			
Absolute value of infarct side	1.1 (3.4)	2.0 (2.6)	0.189
Absolute value of normal side	−0.1 (4.8)	0.9 (4.4)	0.121
Ratio (4 h/1 h of infarct side)	1.1 (0.3)	1.6 (1.1)	0.054
Ratio (Infarct/Normal)	1.1 (0.5)	1.6 (1.3)	0.336

* *p < 0.05*.

## Discussion

This study demonstrated that hydration status may be associated with the progression of infarct volume and the cerebral blood flow after MCAO. Although there was no significant difference between the initial infarct volumes, the volumes of the infarctions among the dehydrated rats were larger at 4th h. Interestingly, dehydration status had a negative impact on cerebral perfusion, which was more prominent in the contralateral normal hemisphere. This finding extended previously reported work that summarized the high prevalence of dehydration among stroke patients (2, 3), the significant impact of dehydration on early neurological deterioration and performance on disability scales (4, 6–8), by adding a physiological basis and imaging evidence. It also supports recent evidence indicating that hydration therapy should be a central feature of acute stroke management (1, 10, 16).

Dehydration and its associated changes in blood viscosity can result in decreased cerebral blood flow (17). Dehydration has been shown to impair cerebral autoregulation following exercise and during a cold pressure test, such that dehydration led to greater changes in mean middle cerebral artery velocity compared with normal hydration status (18, 19). The results of the current study are in line with these studies and provide the first line of evidence suggesting that dehydration status is associated with lower cerebral blood flow, as we observed in the non-infarct healthy hemisphere.

Only few imaging studies have provided imaging evidence regarding the effect of hydration status on the volume of the infarction and cerebral perfusion. In a human study using MRI, we demonstrated that the development of leptomeningeal collaterals after acute MCAO was associated with the patient's hydration status (9). In the current study, we evaluated the volume of infarction and cerebral perfusion with an MCAO rat model using high field MRI to better control for other factors. The reason for an insignificant difference in infarct volume between two groups at the 1st h after MCAO might be due to the pseudo-normalization phenomenon of ADC at early phase of reperfusion (20). Further histological studies are necessary to clarify this.

Our study, nevertheless, had several limitations. First, although we followed the dehydration protocol of previous studies with water deprivation for 48 h, we did not measure the body weight, blood, and urine parameters, such as osmolality and urine-specific gravity, to better quantify the severity of dehydration. Second, the mortality rate of the MCAO rats was very high, especially in the dehydration group. Approximately 50% of the rats died before the first MRI scan had been completed and were excluded from further analysis. This might be due to poor physiological status of the dehydrated rats before and after MCAO. This produces a potential study bias since rats with larger infarctions or more severe decreases in cerebral perfusion might be excluded. Third, this study did not completely fulfill the standard of good laboratory in allocation concealment. Fourth, the rCBF values were relatively low compare to the literatures. This might be due to the partial volume effect, insufficient inversion of the selective pulse, hypocapnia effect as well as noises from the MR system. Furthermore, rats randomized into dehydration group were isolated housed while 2 rats in euhydration group were housed together. This might result in different effect of social isolation.

In conclusion, this preliminary study demonstrated that dehydration status may be associated with the progression of infarct volume and the decrease in cerebral blood flow during the acute stage of ischemic stroke. This preliminary study provided an imaging clue that more intensive hydration therapies and reperfusion strategies are necessary for the management of acute ischemic stroke patients with dehydration status.

## Author contributions

Y-HT: literature review, manuscript writing, tables making; L-CL: study design; I-NL and J-LY: animal study; C-HS and M-YY: imaging collection; Y-CH and H-HW: imaging analysis; J-TY: final manuscript review, and editing.

### Conflict of interest statement

The authors declare that the research was conducted in the absence of any commercial or financial relationships that could be construed as a potential conflict of interest.
