# Supplementary material for: Effects of Dehydration on Brain Perfusion and Infarct Core After Acute Middle Cerebral Artery Occlusion in Rats: Evidence From High-Field Magnetic Resonance Imaging
Source: Front Neurol. 2018 Sep 20;9:786. doi: 10.3389/fneur.2018.00786 (PMC6158308; doi:10.3389/fneur.2018.00786)
Supplement: Supplementary file 1 [file Table_1.DOCX]

| **No.** | **Infarct volume (mm^3^)** | | **1hr ADC (x10^-6^mm^2^/sec)** | | **4hr ADC (x10^-6^mm^2^/sec)** | | **1hr CBF (ml/100g^-1^min^-1^)** | | **4hr CBF (ml/100g^-1^min^-1^)** | |
| --- | --- | --- | --- | --- | --- | --- | --- | --- | --- | --- |
|  | **1h** | **4h** | **infarct side** | **normal side** | **infarct side** | **normal side** | **infarct side** | **normal side** | **infarct side** | **normal side** |
|  | **Dehydration group** | | | | | | | | | |
| **1** | 19.9 | 50.4 | 505.0 | 825.0 | 436.7 | 784.0 | 6.7 | 18.8 | 6.1 | 21.7 |
| **2** | 51.3 | 121.9 | 453.5 | 801.2 | 443.0 | 770.7 | 5.1 | 13.8 | 4.2 | 18.4 |
| **3** | 22.8 | 134.7 | 405.7 | 725.0 | 430.0 | 779.8 | 6.9 | 17.9 | 8.8 | 19.7 |
| **4** | 38.0 | 109.8 | 343.0 | 783.0 | 368.0 | 819.0 | 2.2 | 19.5 | 9.0 | 17.3 |
| **5** | 61.2 | 80.3 | 426.3 | 754.3 | 428.3 | 809.0 | 6.1 | 15.2 | 10.4 | 20.0 |
| **6** | 109.1 | 154.0 | 418.3 | 845.0 | 492.3 | 877.0 | 1.9 | 28.7 | 2.8 | 20.1 |
| **7** | 27.6 | 34.5 | 503.3 | 820.5 | 516.5 | 889.3 | 9.8 | 24.0 | 11.6 | 26.9 |
| **8** | 149.0 | 158.3 | 477.8 | 761.6 | 481.2 | 799.4 | 6.9 | 20.7 | 8.6 | 21.3 |
|  | **Control group** | | | | | | | | | |
| **1** | 58.2 | 88.4 | 529.6 | 883.0 | 618.2 | 911.6 | 6.4 | 21.8 | 6.0 | 21.4 |
| **2** | 29.4 | 37.5 | 322.0 | 813.0 | 389.5 | 803.0 | 16.5 | 20.5 | 10.3 | 24.6 |
| **3** | 57.4 | 62.2 | 433.7 | 761.3 | 428.7 | 803.3 | 7.6 | 24.4 | 3.3 | 20.6 |
| **4** | 23.3 | 28.7 | 451.0 | 848.0 | 503.0 | 803.3 | 15.3 | 24.5 | 13.3 | 24.4 |
| **5** | 24.8 | 29.3 | 468.0 | 846.3 | 440.7 | 815.3 | 6.4 | 34.0 | 6.4 | 27.0 |
| **6** | 50.8 | 64.1 | 474.0 | 820.0 | 449.5 | 806.3 | 15.2 | 30.8 | 18.4 | 20.8 |
| **7** | 45.4 | 63.4 | 459.0 | 752.3 | 432.9 | 791.4 | 13.2 | 26.6 | 15.9 | 26.9 |
| **8** | 17.7 |  | 465.0 | 865.5 |  |  | 16.6 | 33.6 |  |  |
| **9** | 53.3 |  | 466.8 | 883.0 |  |  | 4.5 | 21.8 |  |  |
| **10** | 33.1 |  | 492.3 | 819.7 |  |  | 4.6 | 18.2 |  |  |
