# Supplementary figures and images for: Effects of Dehydration on Brain Perfusion and Infarct Core After Acute Middle Cerebral Artery Occlusion in Rats: Evidence From High-Field Magnetic Resonance Imaging
Source: Front Neurol. 2018 Sep 20;9:786. doi: 10.3389/fneur.2018.00786 (PMC6158308; doi:10.3389/fneur.2018.00786)

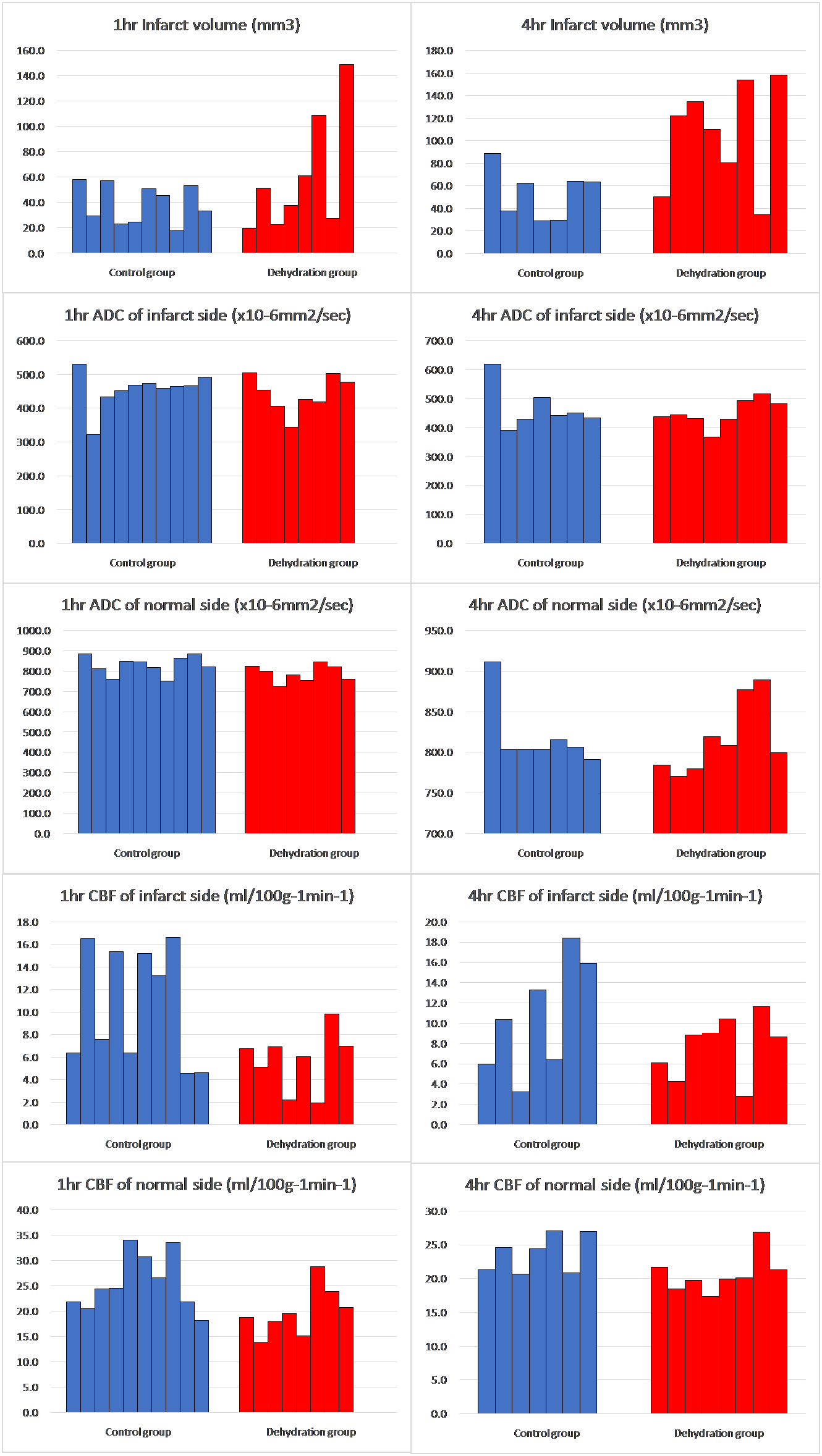

Supplement: Supplementary file 2 [file Image_1.TIF]
